# FuGEFlow: data model and markup language for flow cytometry

**DOI:** 10.1186/1471-2105-10-184

**Published:** 2009-06-16

**Authors:** Yu Qian, Olga Tchuvatkina, Josef Spidlen, Peter Wilkinson, Maura Gasparetto, Andrew R Jones, Frank J Manion, Richard H Scheuermann, Rafick-Pierre Sekaly, Ryan R Brinkman

**Affiliations:** 1 Department of Pathology, University of Texas Southwestern Medical Center, Dallas, TX, USA; 2 Division of Biomedical Informatics, University of Texas Southwestern Medical Center, Dallas, TX, USA; 3 Fox Chase Cancer Center, Philadelphia, PA, USA; 4 Terry Fox Laboratory, BC Cancer Agency, Vancouver, BC, Canada; 5 Centre de recherché du Centre hospitalier de l'Universite de Montreal, Montreal, QC, Canada; 6 Department of Pre-clinical Veterinary Science, Faculty of Veterinary Science, University of Liverpool, Liverpool, UK

## Abstract

**Background:**

Flow cytometry technology is widely used in both health care and research. The rapid expansion of flow cytometry applications has outpaced the development of data storage and analysis tools. Collaborative efforts being taken to eliminate this gap include building common vocabularies and ontologies, designing generic data models, and defining data exchange formats. The Minimum Information about a Flow Cytometry Experiment (MIFlowCyt) standard was recently adopted by the International Society for Advancement of Cytometry. This standard guides researchers on the information that should be included in peer reviewed publications, but it is insufficient for data exchange and integration between computational systems. The Functional Genomics Experiment (FuGE) formalizes common aspects of comprehensive and high throughput experiments across different biological technologies. We have extended FuGE object model to accommodate flow cytometry data and metadata.

**Methods:**

We used the MagicDraw modelling tool to design a UML model (Flow-OM) according to the FuGE extension guidelines and the AndroMDA toolkit to transform the model to a markup language (Flow-ML). We mapped each MIFlowCyt term to either an existing FuGE class or to a new FuGEFlow class. The development environment was validated by comparing the official FuGE XSD to the schema we generated from the FuGE object model using our configuration. After the Flow-OM model was completed, the final version of the Flow-ML was generated and validated against an example MIFlowCyt compliant experiment description.

**Results:**

The extension of FuGE for flow cytometry has resulted in a generic FuGE-compliant data model (FuGEFlow), which accommodates and links together all information required by MIFlowCyt. The FuGEFlow model can be used to build software and databases using FuGE software toolkits to facilitate automated exchange and manipulation of potentially large flow cytometry experimental data sets. Additional project documentation, including reusable design patterns and a guide for setting up a development environment, was contributed back to the FuGE project.

**Conclusion:**

We have shown that an extension of FuGE can be used to transform minimum information requirements in natural language to markup language in XML. Extending FuGE required significant effort, but in our experiences the benefits outweighed the costs. The FuGEFlow is expected to play a central role in describing flow cytometry experiments and ultimately facilitating data exchange including public flow cytometry repositories currently under development.

## Correspondence

Flow cytometry (FCM) experiments need to be described and recorded in a standardized way to allow not only correct interpretation of experiment design, but also consistent data archiving and sharing. To solve this problem we designed a MIFlowCyt-compliant data model and a markup language for data exchange and integration between computational systems.

### Extending FuGE for Flow Cytometry

FuGE [1] is an extensible framework for standards in functional genomics. Its core model consists of a set of generic object classes representing the common information in different laboratory workflows and experimental pipelines. FuGE provides numerous extension points and has been adopted by proteomics, genomics, and metabolomics standards bodies. Using and extending FuGE to capture experimental information facilitates data integration and sharing among different communities. FuGEFlow is the extension of FuGE for FCM experiments.

We recommend domain developers interested in extending FuGE start from documentation available from the FuGE Website [2] and published work [1,3], paying careful attention to the extension guidelines and recommendations and communicating with the FuGE development community through discussion forums and email lists. While extending FuGE requires a thorough familiarity with the FuGE development infrastructure, our experience suggests the benefits, particularly the potential cross-platform data integration, are worth the learning cost. The reuse of core FuGE classes makes FuGEFlow flexible enough to accommodate FCM data from workflows we did not anticipate during the modelling process, and allows FuGEFlow to be consistent and interoperable with other FuGE-compliant data models. To ease the integration among different FuGE extensions, coordination of cross-domain efforts is also important. For example, FuGE has only one generic material class, while other common concepts like organism are not included in FuGE v1. If different domain developers create their own organism classes with different data elements, integrating organism data across these domains will be challenging. To address this issue, FuGE provides a design patterns page for developers to share their class diagrams so that particular design paradigms can be reused. As the FuGE community grows, this resource will become a useful addition to the FuGE modelling documentation.

### Accommodation of MIFlowCyt

Table 1 illustrates relationships between high level MIFlowCyt [4] terms and FuGEFlow classes. For a more detailed mapping, please refer to the Additional file 1. The resulting UML data model Flow-OM and XML schema Flow-ML can be found in Additional files 2 and 3.

**Table 1:** The relationships between high level MIFlowCyt terms and FuGEFlow classes

MIFlowCyt term		FuGEFlow class
Experiment Overview	1.1 Purpose	FuGE::Bio::Investigation::HigherLevelAnalysis
	
	1.2 Keywords	FuGE::Bio::Investigation::Investigation
	
	1.3 Experiment Variables	FuGE::Bio::Investigation::Investigation
	
	1.4 Organization	FuGE::Common::Audit::ContactRole
	
	1.5 Primary Contact	FuGE::Common::Audit::ContactRole
	
	1.6 Date	FuGE::Bio::Investigation::Investigation
	
	1.7 Conclusion	FuGE::Bio::Investigation::HigherLevelAnalysis
	
	1.8 Quality Control Measures	FuGE::Bio::Investigation::InvestigationComponent
	
	1.9 Other Relevant Experiment Information	FuGE::Bio::Investigation::Investigation

Specimen Details	2.1 Specimen Material Description	FCM::Sample; FCM::Organism
	
	2.2 Sample Treatment(s) Description	FuGE::Common::Protocol::GenericProtoclApplication
	
	2.3 Fluorescence Reagent Description	FCM::FluorescentReagent

Instrument Details	3.1 Manufacturer	FuGE::Equipment; FCM::Cytometer
	
	3.2 Model	FuGE::Equipment; FCM::Cytometer
	
	3.3 Configuration and Settings	FuGE::Equipment
	
	3.4 Other Relevant Instrument Settings	FCM::Cytometer

Data Analysis Details	4.1 List-Mode Data File	FCM::ListModeDataFile
	
	4.2 Compensation Details	FCM::CompensationProtocol
	
	4.3 Gating (Data Filtering) Details	FCM::DataFilteringProtocol
	
	4.4 Data Transformation Details	FCM::DataTransformationProtocol

**Figure 1:**
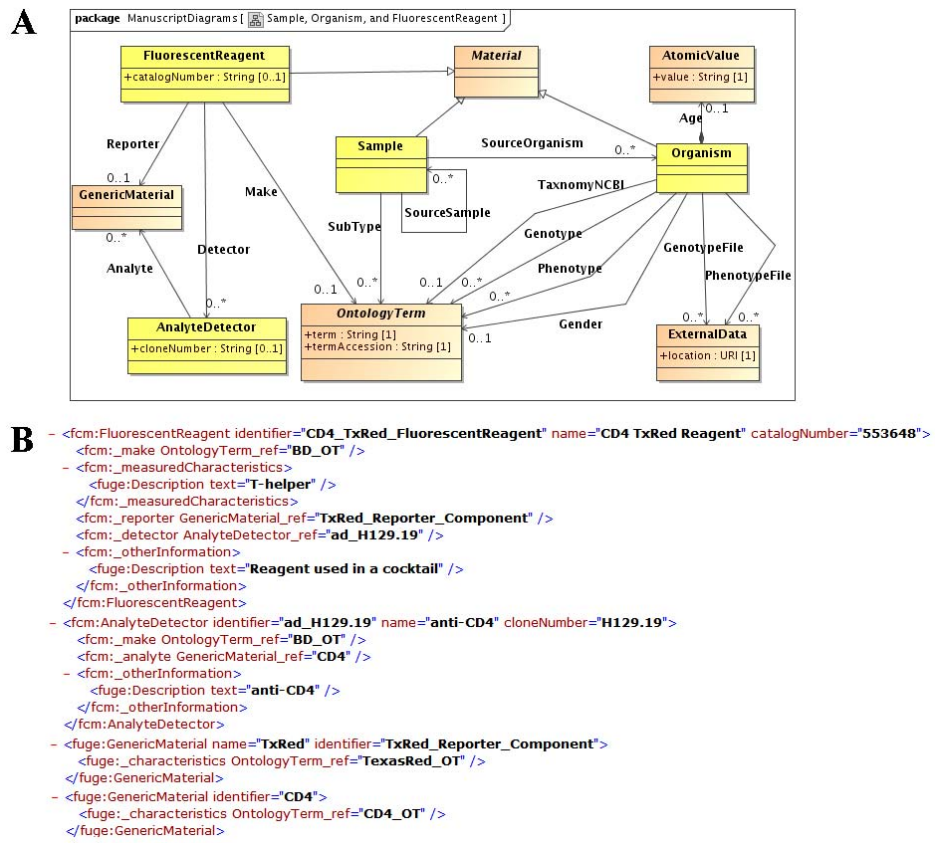
**Extending FuGE classes to model Sample, Organism, and Fluorescent Reagent**. Figure 1A shows FuGE classes in light brown and Flow-OM classes in bright yellow. The *Organism *class exemplifies a non FCM-specific concept common across a wide range of biological domains. *Organism *inherits identification and other generic properties, as well as the ability to be consumed and produced in a protocol application, from the FuGE *Material *class. Organism properties defined in the MIFlowCyt, such as taxonomy, gender, and age are modelled explicitly as references to the *OntologyTerm*, *Description *and *AtomicValue *classes. *ExternalData *is used to reference files outside of the scope of the model, in this case phenotype and genotype files of the *Organism*. The *FluorescentReagent *class is an example of a composite biological material. *FluorescentReagent *combines reporter (*GenericMaterial*), detector (*AnalyteDetector*) and a list of analytes (*GenericMaterial*) into a single biological material. Figure 1B shows the corresponding Flow-ML representation of a fluorescent reagent. In FCM experiments a fluorescent reagent usually consists of an analyte detector (antibody) and a reporter (fluorescence marker). An analyte detector is a common entity in various biological experiments and is described by the analytes (antigens) it binds to. In this example, reagent CD4_TxRed consists of the reporter TxRed and the analyte detector anti-CD4. The analyte detector anti-CD4 binds with the analyte CD4.

**Figure 2:**
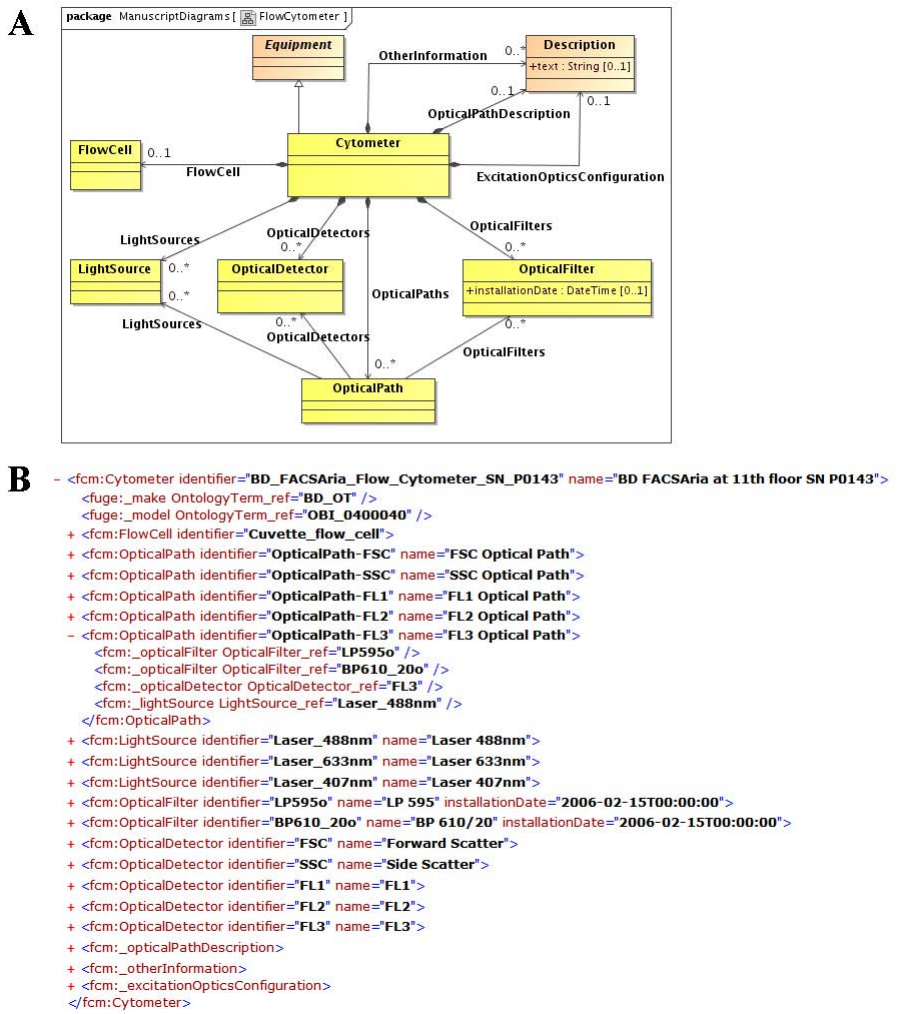
**Extending FuGE classes to Model a Flow Cytometer**. Figure 2A shows a flow cytometer in Flow-OM. *Cytometer *and its components (i.e., *FlowCell*, *OpticalDetector*, *OpticalFilter *and *LightSource*) are extended from *Equipment*. Besides physical components, Flow-OM describes *OpticalPath *containing sequences of light sources, optical detectors, and filters. Figure 2B shows an example flow cytometer described in Flow-ML. In this example, flow cytometer contains one flow cell, three lasers, and a few other optical components.

The experiment overview category in MIFlowCyt is the most generic and each term in this category was mapped to existing FuGE classes. We created new classes inherited from the FuGE *Material *class to describe FCM experimental materials that typically include samples, organisms, and fluorescent reagents (Figure 1). To capture instrument details, we extended the FuGE *Equipment *class to model a flow cytometer and each of its components, such as a flow cell, a light source, an optical detector, and an optical filter (Figure 2). To describe FCM data analysis we created a new *ListModeDataFile *class to reference the Flow Cytometry Standard (FCS, [5]), a well established format describing raw FCM data. We used the FuGE *ExternalData *class to reference Gating-ML [6] documents describing data modifications.

While modelling MIFlowCyt, we realized the granularity of information in the data model is very important. Some information needs to be explicitly listed in the model as attributes, while other information can be archived as binary files or in text descriptions. For each case, the decision was made based on whether the information will need to be computationally processed or used in discrete form. For example, voltage of an optical detector of a flow cytometer can usually be adjusted by users (e.g., calibrating background with non-stained controls) to significantly alter output data. Its value might interest other users and therefore is explicitly modelled as a *ParameterValue *to associate with *OpticalDetectorApplication*. In contrast, individual excitation optics configurations are rarely modified and not expected to be computationally processed and thus they are modelled as free text description of the *Cytometer*.

To ensure FuGEFlow was sufficient to capture the FCM experimental details required by MIFlowCyt, we manually encoded a MIFlowCyt-compliant example dataset into Flow-ML format. The example data set contains a complete FCM experiment on peripheral blood (PB) cells of transplanted mice to study how hematopoietic stem cell (HSC) from the donor contributes to the phenotypes of the lymphocytes and myeloid cells of the host. The data set includes all details required by MIFlowCyt. The example dataset and the resulting XML can be found in Additional files 4 and 5.

### Interplay with Other FCM Standards

Instead of remodelling information contained in other data formats, FuGEFlow is designed to reuse and integrate with existing standards. For example, the FCS data standard is supported by all analytical instrument and third party software suppliers for the exchange of the fluorescent signals captured by cytometers. FuGEFlow simply references FCS data files without any duplication of the captured fluorescence intensity values. Similarly, Gating-ML files are referenced to encode the description of gates and other data transformations. This XML format contains details about data transformations including compensation (subtraction of the fluorescence due to overlap of the emission spectra) and gating (filtering of the dataset based on characteristics of its members). One could also reference other types of external data files. The readers are encouraged to refer to Additional files 5 and 6 for Gating-ML example and details on how it is referenced from Flow-ML.

CytometryML [7] represents another FCM-related file format, which is also one of the earliest attempts based on DICOM to describe FCM and image cytometry data with XML. There are a couple of differences between Flow-ML and Cytometry-ML. First, Cytometry-ML defines static standalone concepts, while Flow-ML extends from FuGE that models protocols that link standalone components to descriptive experimental workflows. Second, Cytometry-ML defines in its own terms concepts reaching from primitive data types to descriptions of FCM instruments and data including periodic tables of elements, lists of organs, enumerations of cell types, and definitions of scientific units. Flow-ML takes an alternative approach by allowing existing FCM standards such as FCS, Gating-ML, ontologies, and even CytometryML to be referenced in a Flow-ML file. This makes Flow-ML a complementary format to existing standards and increases its potential of being adopted by FCM community.

### Software Implementations

FuGEFlow compliant data can be captured by the FuGE toolkit [8] which generates basic software interface and applications for storing and searching data from FuGE model or its extension. There is also an implementation to convert Flow-ML into a tab-delimited format. From the user's perspective, managing data in tab-delimited spreadsheet-based format may be more convenient than working with XML [9]. The Investigation, Study, Assay tab-delimited format (ISA-TAB, [10]) is a general purpose framework that aims to design and use tabular formats to communicate both metadata and data from different omics-based experiments. The EBI's BioInvestigation Index project used ISA-TAB format to create common structured representation and storage mechanism for a variety of biological, biomedical and environmental studies. Flow-ML data can be converted to ISA-TAB through a XSL transformation stylesheet. The example stylesheet transforming Additional File 5 to ISA-TAB HTML can be found in Additional File 7.

We anticipate that a number of software implementations of Flow-ML will appear in near future. We are currently building a public FCM data repository based on Flow-ML for peer-reviewed publications, and planning integration strategy between Flow-ML and existing FCM-related databases such as ImmPort. As FuGE is being adopted by more and more communities, we believe FuGEFlow will play a central role in future FCM informatics.

## Abbreviations

DICOM: Digital Imaging and Communications in Medicine; HTML: HyperText Markup Language; UML: Unified Modelling Language; XML: Extensible Markup Language; XSD: XML Schema Definition; XSL: Extensible Stylesheet Language.

## Authors' contributions

RRB conceived of the project, guided its development and helped revise the manuscript. YQ, OT, JS, and PW developed FuGEFlow. YQ and OT drafted the manuscript. MG provided example data and joined discussion. JS encoded the example data and helped revise the manuscript. PW documented FuGEFlow and helped revise the manuscript. ARJ helped develop FuGEFlow, reviewed and revised the manuscript. RHS, R-PS, and FJM provided valuable advice in both model development and manuscript revision. All authors read and approved the final manuscript.

## Supplementary Material

Additional file 1 **Mapping between MIFlowCyt and FuGEFlow**. Mapping between MIFlowCyt terms and FuGEFlow classes and attributes. Click here for file

Additional file 2 **Flow-OM UML model**. The Flow-OM data model in UML.
MagicDraw Community edition is the recommended viewer : Click here for file

Additional file 3 **Flow-ML markup language**. The Flow-ML markup language described as XML schema. Click here for file

Additional file 4 **MIFlowCyt-compliant experiment in plain English**. Example of MIFlowCyt-compliant data described in plain English. Click here for file

Additional file 5 **MIFlowCyt-compliant experiment in Flow-ML**. Example of MIFlowCyt-compliant data described in Flow-ML. Click here for file

Additional file 6 **MIFlowCyt-compliant gating in GatingML**. Gating-ML file is referenced from the MIFlowCyt-compliant data example. Click here for file

Additional file 7 **Flow-ML data in ISA-TAB**. FuGEFlowtoISATAB.xsl stylesheet transformation file creates ISA-TAB HTML representation of Flow-ML data. Click here for file
