# Supplementary material for: FuGEFlow: data model and markup language for flow cytometry
Source: BMC Bioinformatics. 2009 Jun 16;10:184. doi: 10.1186/1471-2105-10-184 (PMC2711079; doi:10.1186/1471-2105-10-184)
Supplement: Additional file 4 — MIFlowCyt-compliant experiment in plain English. Example of MIFlowCyt-compliant data described in plain English. [file 1471-2105-10-184-S4.pdf]

Minimum Information about a Flow Cytometry Experiment (MIFlowCyt)

Experiment Annotation Example

Maura Gasparetto, Josef Spidlen, Ryan R. Brinkman
Terry Fox Laboratory, BC Cancer Research Centre
Vancouver, BC, Canada

Acknowledgement

This work has been supported by NIH grant EB005034 from the National Institute of Biomedical Imaging And Bioengineering and by the NIAID Bioinformatics Integration Support Contract AI40076 (BISC). The content is solely the responsibility of the authors and does not necessarily represent the official views of the National Institute of Biomedical Imaging And Bioengineering or the National Institutes of Health.

The work may be used under the terms of the *Creative Commons Attribution-ShareAlike 3.0 Unported* license. You are free to share (copy, distribute and transmit), and adapt the work under the conditions specified at http://creativecommons.org/licenses/by-sa/3.0/legalcode.

June 27th, 2008

Introduction

This document represents an example description of a flow cytometry experiment that is compliant with the Minimum Information about a Flow Cytometry Experiment (MIFlowCyt).

MIFlowCyt has been reviewed by members of the International Society for Analytical Cytology (ISAC) and other interested parties and has been endorsed by the ISAC President and ISAC Council as an ISAC Recommendation. It is a stable document and may be used as reference material. The purpose of MIFlowCyt is to establish criteria to record flow cytometry experiments in a way that provides enough detail to allow for correct interpretation of experimental details including samples, analysis and results. MIFlowCyt promotes consistent annotation of biological and technical issues surrounding a flow cytometry experiment by specifying the requirements for data content and by providing a structured framework for capturing information. This example is intended for the purpose of demonstrating MIFlowCyt only.

A MIFlowCyt-compliant flow cytometry experiment description shall include all relevant information specified in the MIFlowCyt standard. MIFlowCyt states the content of the provided information; it does not imply the format of the information or whether an item should be directly provided or referenced. Within this example we follow the MIFlowCyt structure in order to demonstrate MIFlowCyt as clearly as possible.

1. Experiment Overview

1.1. Purpose

The purpose of the experiment is to quantify donor hematopoietic stem cells (HSC) contribution to lymphocytes and myeloid cells by flow cytometric analysis of peripheral blood (PB) cells stained with appropriate combinations of donor specific marker and lineage specific antibodies.

1.2. Keywords

Blood, transplant, donor, recipient, leukocyte, lymphocyte, monocyte, granulocyte

1.3. Experiment Variables

- Number of transplanted HSC cells

Decreasing numbers of donor HSC cells (5, 20, 100) were injected at day 0 into 3 groups respectively of recipient mice; 3 mice per group. Kit^{+}/sca-1^{+}/$Lineage^{-}$ (KSL) HSC cells (500) were injected into each mouse of the control group (3 mice in the control group).

1.4. Organization

1.4.1. Name: BC Cancer Research Centre

1.4.2. Address: 675 West 10th Avenue, Vancouver, BC, V5Z 1L3, Canada

1.5. Primary Contact

1.5.1. Name: Clayton Smith

1.5.2. Email: clsmith@bccancer.bc.ca

1.6. Date

Transplantation performed on July 28, 2006.

Flow cytometry analysis performed 4, 8, and 16 weeks post transplantation.

1.7. Conclusions

- 1 mouse out of 3 in the 5 cell group was considered positive
- 1 mouse out of 3 in the 20 cell group was considered positive
- 3 mouse out of 3 in the 100 cell group were considered positive
- 3 mouse out of 3 in the 500 cell control group were considered positive

Mice are considered reconstituted (positive) if their PB contains ≥1% of donor derived myeloid and lymphoid cells at 4 months or later post transplantation.

1.8. Quality Control Measures

PB has been collected from positive as well as negative mice in order to provide a staining control. Mice from the 500 KSL group were consider as the positive controls; mice that are not reconstituted as the negative controls.

Unstained controls have not been used as they are not critical for the evaluation of results because in each sample dull negative cells are being compared to bright positive populations.

1.9. Other Relevant Experiment Information

The Competitive Repopulation Assay is used for the experiment. It is a quantitative assay for long-term repopulating stem cells with the potential for reconstituting all hematopoietic lineages. This assay has two key features. The first is the use of competitive repopulation conditions that ensure not only the detection of a very primitive class of hematopoietic stem cells but also the survival of lethally irradiated mice transplanted with very low numbers of such cells. The second is the use of a limiting-dilution experimental design to allow stem cell quantitation.

- Szilvassy SJ, Humphries RK, Lansdorp PM, Eaves AC, Eaves CJ. Quantitative Assay for Totipotent Reconstituting Hematopoietic Stem Cells by a Competitive Repopulation Strategy. *Proceedings of the National Academy of Sciences*, 1990, Vol 87, 8736-8740. PMID: 2247442

2. Flow Sample/Specimen Details

2.1. Sample/Specimen Material Description

2.1.1. Biological Samples

2.1.1.1. Biological Sample Description: 50-75µl of PB collected in heparinized capillary tubes and placed in 12x75 FACS tubes for staining.

2.1.1.2. Biological Sample Source Description: Mus musculus. C57BL/6J:Pep3b; C57BL/6J:W^{41}/W^{41}

2.1.1.3. Biological Sample Source Organism Description: 50-75µl of PB collected in heparinized capillary tubes and placed in 12x75 FACS tubes for staining.

- *Taxonomy*: *Mus musculus* donor: C57BL/6J:Pep3b; recipient: C57BL/6J:W^{41}/W^{41}
- *Age*: 6 months, specifically, 4 donor mice born on January 6, and 12 recipient mice born between January 24 and February 4, 2006.
- *Gender*: male (all mice)
- *Phenotype*: donor: black Pep3b mice; recipient: white W^{41}/W^{41} mice
- *Genotype*: Ly-5 congenic C57BL/6 mouse strains (histologically compatible but genetically distinguishable) in combination of W^{41}/W^{41} in recipient mice, which are bearing mutation in c-kit.
- *Treatment*:
  - *Irradiation*: July 28, 2006 at 5-5:30pm; dose: 360 rads (all recipient mice)
  - *Injected test cells*: 5, 20 and 100 donor cells were respectively injected into each of 3 recipient mice per group.
  - *Injected control cells*: 500 KSL HSC were injected into each mouse of the control group.

  Sublethal irradiation preconditioning in c-kit mutant (W^{41}/W^{41}) mice produces long-term engraftment with minimal morbidity and mortality. CRU detection, that involves transplanting decreasing numbers of "test" cells, is easier to assess because reconstitution by single HSCs is very dominant in this setting.
- *Other Relevant Biological Sample Source Organism Information:* Animals located at the Animal Resource Centre (ARC) of the BC Cancer Research Centre (BCCRC)

2.1.1.4. Other Relevant Biological Sample Information: Cage number and mouse ID have been used as sample/file identifier.

2.1.2. Environmental Samples

N/A

2.1.3. Other Samples

N/A

2.2. Sample Characteristics

Expected/analyzed types of cells: red blood cells, lymphoid and myeloid properties, Ly-5.1 vs. Ly-5.2. After lysing red blood cells, samples are stained for lymphoid and myeloid properties in addition to the specific form (Ly-5.1 or Ly-5.2) of the alloantigen ptprc (protein tyrosine phosphatase receptor type c polypeptide) to detect donor and recipient respectively.

2.3. Sample Treatment Description

- Single cell suspensions are prepared and the cell concentrations are adjusted to 107/ml.
- Block Immunoglobulin Fc Receptors: In the mouse system, purified antibodies directed against mouse FcγII/III (Mouse BD FcBlock™, CD16/CD32, catalogue no. 553141 and 553142) have been used to block nonspecific staining due to FcR. To block FcR with BD FcBlock reagents, the cells are preincubated with 10 µg/ml of BD FcBlock antibody per 2x107 cells for 15–20 min at 4°C. The cells are not washed before the first staining step.
- Cells have been incubated for 40 minutes at 4°C in a staining buffer (approx. 106 cells in 100µl of staining buffer). The staining buffer contained a pre-titrated, optimal concentration (≤ 1µg) of a fluorescent monoclonal antibody specific for a receptor or with an immunoglobulin (Ig) isotype-matched control respectively (see below for details).
- After the incubation, cells have been washed 1x with 2ml of staining buffer and pelleted by centrifugation (250 X g for 5 min); supernatant has been removed.
- Finally, cells have been resuspended in PBS/2%FCS (Phosphate Buffered Solution, Fetal Calf Serum) for flow cytometric analysis.

2.4. Fluorescence Reagent Description

Each sample has been stained according to the following table:

Optical detector	*FL1*	*FL2*	*FL3*	*FL4*	*FL6*	*FL8*
Reporter	*FITC*	*PE*	*Tx-Red*	*PerCP*	*APC*	*DAPI*
Sample	+Ab	+Ab	+Ab	+Ab	+Ab	-

Compensation tubes have been created as follows:

Optical detector	*FL1*	*FL2*	*FL3*	*FL4*	*FL6*
Reporter	*FITC*	*PE*	*Tx-Red*	*PerCP*	*APC*
Tube #1	+Ab	-	-	-	-
Tube #2	-	+Ab	-	-	-
Tube #3	-	-	+Ab	-	-
Tube #4	-	-	-	+Ab	-
Tube #5	-	-	-	-	+Ab

The following reagents are being used:

Characteristic	*Analyte*	*Detector*	*Reporter*	*Manufact.*	*Clone*	*Cat#*
T helper	CD4	Anti-CD4	Tx-Red	BD	H129.19	553648

T suppressor	CD8	Anti-CD8	Tx-Red	BD	53-6.7	553028
B cells	CD45R	Anti-CD45R	APC	BD	RA3-6B2	553092
Granulocytes	Gr1	Anti-Gr1	PE	BD	1A8	551461
Monocytes	CD11b	Anti-CD11b	PE	BD	M1/70	557397
Donor leukocytes	CD45.1	Anti-CD45.1	FITC	BD	A20	553775
Recipient leukocytes	CD45.2	Anti-CD45.2	PerCP	BD	104	552950
Cell viability	DNA (membrane-compromised cells)	DAPI	DAPI	Exalpha	N/A	7034

3. Instrument Details

3.1. Instrument Manufacturer

BD Biosciences

http://www.bdbiosciences.com/home/

3.2. Instrument Model

BD FACSAria™ Flow Cytometer.

http://www.bdbiosciences.com/immunocytometry_systems/products/display_product.php?keyID=53

Serial number P0143

Technical specification at

http://www.bdbiosciences.com/pdfs/whitePapers/23-8385-00.pdf

3.3. Instrument Configuration and Settings

3.3.1. Flow Cell and Fluidics

The instrument has not been altered; fixed-alignment cuvette flow cell.

3.3.2. Light Sources

The instrument has not been altered; three-laser base configuration with ACDU.

- 488-nm Coherent® Sapphire™ solid state; 14mW;
- 633-nm JDS Uniphase™ HeNe air-cooled; 14mW;
- 407-nm Point Source Violet solid state; 12mW;

3.3.3. Excitation Optics Configuration

The instrument has not been altered.

3.3.4. Optical Filters

The instrument has not been altered, all filters are original and came with the instrument (February 15, 2007). See also figure below.

3.3.5. Optical Detectors

The instrument has not been altered. Detector voltages have been set to: FSC=220V; SSC=330V; FL1=500V; FL2=500V; FL3=500V; FL4=500V; FL6=500V; FL8=550V.

3.3.6. Optical Paths

The instrument has not been altered. The following figure shows the filter and detector configuration:

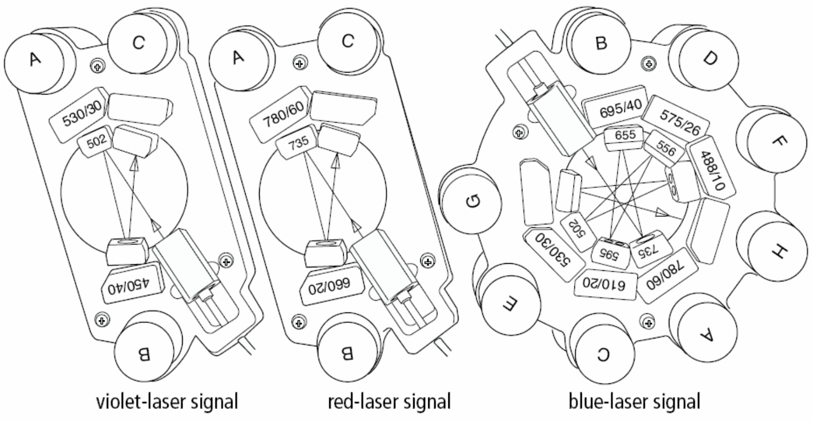

Figure 1: Optical detectors configuration

Laser 488nm: FL1 – PMT E; FL2 – PMT D; FL3 – PMT C; FL4 – PMT B;

Laser 633nm: FL6 – PMT B;Laser 407nm: FL8 – PMT B;

Detector Array (Laser)	PMT	LP Mirror	BP Filter	Intended Dye
Octagon (488-nm blue laser)	A	735	780/60	PE-Cy7
	B	655	695/40<br>675/20	PerCP-Cy5.5 or PI<br>PerCP alone
	C	595	610/20	PE-Texas Red
	D	556	575/26<br>585/42	PE or PI<br>Alternative for PE/PI when not using PE-Texas Red
	E	502	530/30	FITC
	F	—	488/10	Side scatter (SSC)
Trigon (633-nm red laser)	A	735	780/60	APC-Cy7
	B	—	660/20	APC
Trigon (407-nm violet laser)	A	502	530/30	Alexa Fluor 430, Hoeschst, DAPI
	B	—	450/40	Cascade blue, Pacific blue, Alexa Fluor 405

Figure 2: Optical detectors configuration details

3.4. Other Relevant Instrument Details

- BD FACSAria User's Guide: http://ncxt.lbl.gov/files/lab/BD_Aria_manual.pdf
- BD FACSDiva Software Reference Manual: http://facs.stanford.edu/sff/doc/bdfacsdivasoftwareuserguide.pdf

4. Data Analysis Details

4.1. List-mode Data Files

FCS data files can be obtained by contacting Dr. Clayton Smith after this work has been published.

4.2. Compensation Description

Compensation has been performed computationally post acquisition according to the following spillover matrix (values in %):

	FL1-A	*FL2-A*	*FL3-A*	*FL4-A*	*FL5-A*	*FL6-A*	*FL7-A*	*FL8-A*
FL1-A	100	20	4	0	0	0	0	0
FL2-A	0.7	100	23	2.9	0	0	0	0
FL3-A	0	18	100	25	2	0	0	0
FL4-A	0	0	0	100	12	0	0	0
FL5-A	0	25	5	0.8	100	0	0	0
FL6-A	0	0	3	0	0	100	0	0
FL7-A	0	0	0	0	0	0	100	0
FL8-A	0	0	0	0	0	0	0	100

4.3. Data Transformation Details

4.3.1. Purpose of Data Transformation

Visualization and gating.

4.3.2. Data Transformation Description

FlowJo default visualization settings have been used for gating:

- Select which parameters to display with logarithmic scaling: All fluorescence parameters
- Linear channel number corresponding to left edge of log display: 3
- Number of decades to display log-converted data: 5 for pulse area parameters, 4 for pulse height parameters
- Logicle transformation enabled: number of decades: 4.5, additional negative display size: 0, width basis: -10.

4.3.3. Other Relevant Data Transformation Details

FlowJo version 6.4.7. on a Mackintosh OSX 10.2.3 has been used for transforming the data.

4.4. Gating (Data Filtering) Details

The same gating strategy has been used for all data files (all recipient mice at different time points post transplant). All these gates would be reported in a real experiment description (e.g., attached as Gating-ML descriptions or as external image files). In order to keep this document as a clear and simple example we provide details only on gating of a single list mode data file and we include these as images within this document.

4.4.1. Gate Description

The gating strategy involves the following gates:

- FSC-SSC gate to define the leukocytes (Figure 3).
- FSC-DAPI gate applied on leukocytes to discriminate viable leukocytes (Figure 4).
- CD45.1-CD45.2 gates applied on the viable leukocytes for detecting the donor and the recipient percentage of engraftment respectively (Figure 5).
- Gr-1/CD11b-B220 gate applied on donor's viable leukocytes to determine the type of lineage reconstitution. After dividing the PE/APC plot into four quadrants, granulocytes/monocytes and lymphocytes (B cells) would appear in the upper left quadrant and lower right quadrant respectively (Figure 6).
- B220-CD4/CD8 gate applied on donor's viable leukocytes to determine the type of lineage reconstitution. After dividing the TxRed/APC plot into four quadrants, T-lymphocytes and B-lymphocytes would appear in the upper left quadrant and lower right quadrant respectively (Figure 7). A different combination of two stains and two-color analysis is required to gate multi-color subsets of leukocytes in the same staining protocol. (Note: In this gate the B220 % is smaller (29.4) than in the previous gate as the double positive events (1.64%) are not included.)

4.4.2. Gate Statistics

The following table shows percentages of each of the subpopulations defined by described gates.

ID#100B	SSC-FSC	Viable	Recipient	Donor	B cells	T cells	Myeloid
Tot %	55.6						
SSC-FSC %		93.6					
Viable %			55.5	43.1			
Donor %					30.9	30.4	38.8

Gate Statistics would be reported for all list mode data files on which gates have been applied in a real experiment description. Again, we are focusing on a single data file only in order to keep this document as a clear and simple example.

4.4.3. Gate Boundaries

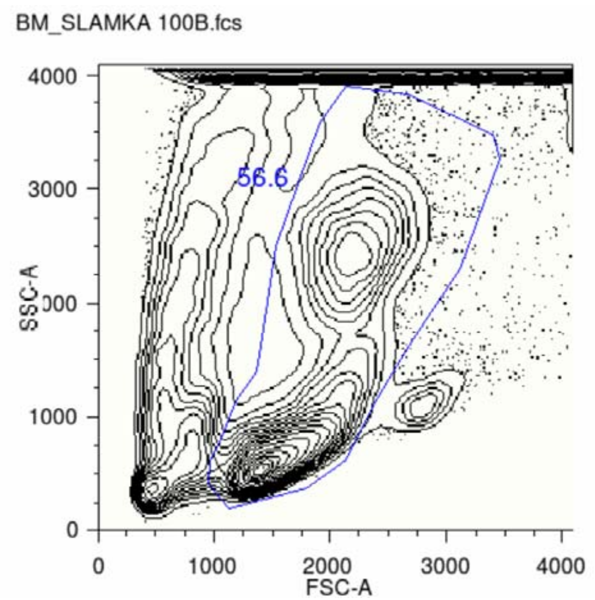

Figure 3: FSC-SSC gate to define the leukocytes

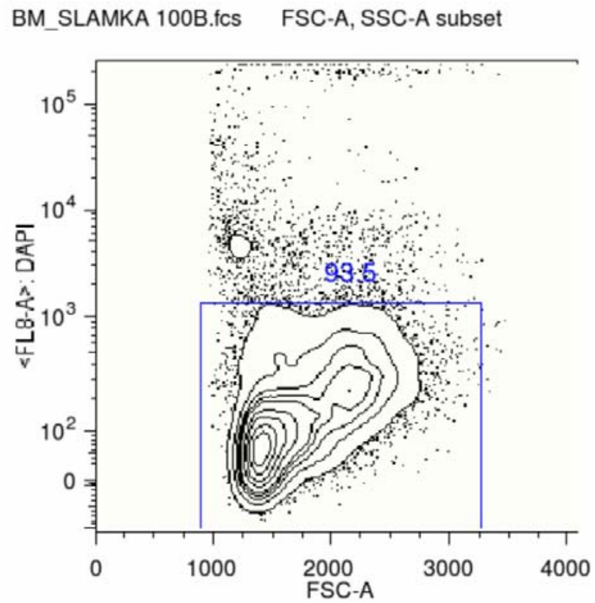

Figure 4: FSC-DAPI viable gate

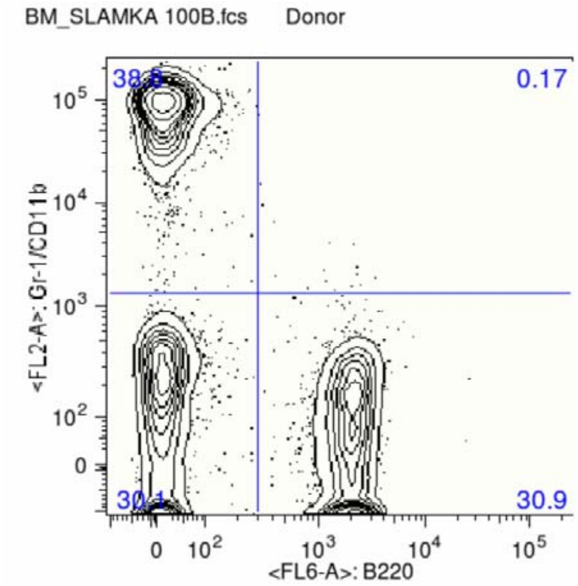

Figure 5: Gr1/CD11b-B220 gate

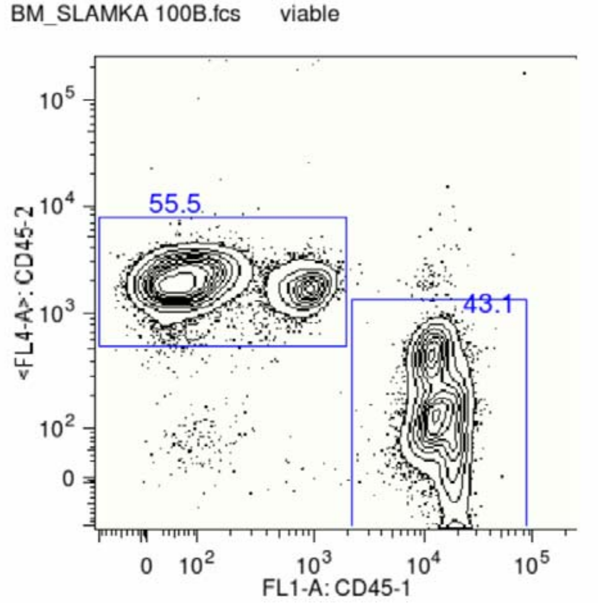

Figure 6: CD45.1-CD45.2 gates

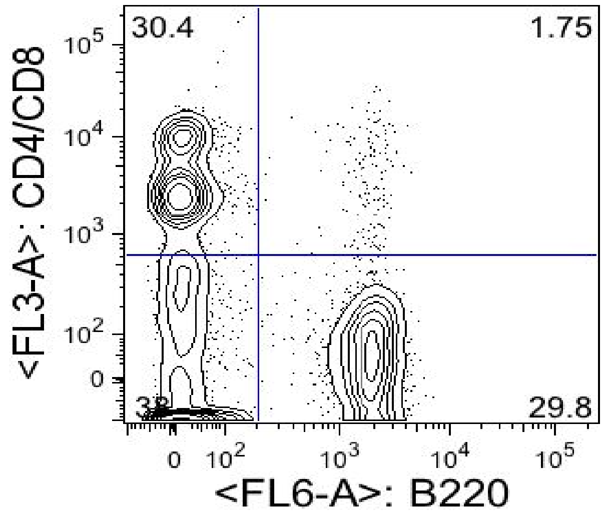

Figure 7: B220-CD4/CD8 gate

4.4.4. Other Relevant Gate Information

For example, FlowJo workspace files could be included to provide additional gate information if this was a real experiment description.
